# Seafood Intake as a Method of Non-Communicable Diseases (NCD) Prevention in Adults

**DOI:** 10.3390/nu13051422

**Published:** 2021-04-23

**Authors:** Dominika Jamioł-Milc, Jowita Biernawska, Magdalena Liput, Laura Stachowska, Zdzisław Domiszewski

**Affiliations:** 1 Department of Human Nutrition and Metabolomics, Pomeranian Medical University in Szczecin, 71-460 Szczecin, Poland; 2 Department of Anaesthesiology and Intensive Therapy, Pomeranian Medical University, 71-242 Szczecin, Poland; jowita.biernawska@pum.edu.pl; 3 Department of Pharmacognosy and Natural Medicines, Pomeranian Medical University in Szczecin, 70-111 Szczecin, Poland; mliput@pum.edu.pl; 4 Department of Biochemical Sciences, Pomeranian Medical University in Szczecin, 71-460 Szczecin, Poland; laura.stachowska@pum.edu.pl; 5 Department of Food Industry Processes and Facilities, Koszalin University of Technology, 75-620 Koszalin, Poland; zdzislaw.domiszewski@tu.koszalin.pl

**Keywords:** diet, seafood, elderly people, health, non-communicable diseases

## Abstract

Seafood (fish in particular) is one of the main food groups in nutrition models with proven health benefits. Seafood has long been considered a very valuable dietary component, mainly due to presence of *n*-3 polyunsaturated fatty acids (*n*-3 PUFA) but it is also an important source of protein (including collagen), anserine, taurine, iodine, selenium, vitamin A, vitamin K, vitamin D, tocopherols, B vitamins and astaxanthin. Considering the beneficial effects of these ingredients on blood pressure, lipid profile and the inflammatory process, seafood should be an essential component of the diet. Non-communicable diseases (NCD) such as cardiovascular diseases, cancer, diabetes and mental disorder, chronic respiratory diseases are common diseases associated with advanced age. Promotion of a healthy lifestyle (including proper nutritional behavior) and prevention of diseases are the most effective and efficient ways to decrease premature mortality from NCD and to maintain mental health and well-being. This review article shows the potential preventive and therapeutic effects of seafood with an emphasis on fish. Our narrative review presents the results of systematic reviews and meta-analysis.

## 1. Introduction

Seafood includes fish or shellfish, divided into crustaceans and molluscs. Crustaceans include shrimp, lobsters, crabs, crayfish, and molluscs include scallops, oysters, clams, and squid [1]. The most important national and international nutritional guidelines recommend the regular consumption of fish [2,3]. Seafood (fish in particular) is one of the main food groups in nutrition models with proven with health benefits, such as the Mediterranean diet [4] and Dietary Approaches to Stop Hypertension dietary pattern [5].

The consumption of fish in the USA has not changed over almost two decades (1999–2016) [6]. There are variations in the amount of fish consumed both within and between populations. Consumption of fish in many Western populations differs. It can be divided in three groups regarding the frequency of consumption and each group is represented by approximately one third of the studied populations.

The first group includes people who do not eat fish at all, the second group includes people who consume fish up to once a week, and the representatives of the third group eat fish more often than once a week. The differences in fish consumption are undoubtedly due to personal and environmental factors (e.g., culture, place of residence, family habits, socioeconomic status) [7]. In the European and US populations surveyed (86,467 participants from the US, Estonia, Finland, Greece, Italy, and the Netherlands), the average fish consumption ranged from 0.19 servings/day (19 g/day) to 0.75 servings/day (75 g/day). Overall, participants in the European cohorts had a higher fish consumption than the US cohorts [8]. It has been estimated approximately 37% of elderly (range 72–83 years) eat one serving of fish less than once a week, 29% 1 serving/weekly, approx. 27% 2–3 servings/weekly, and only 6% more than four servings/weekly (results from one French cohort and four US cohorts) [9].

The mean consumption of eicosapentaenoic acid and docosahexaenoic acid (EPA and DHA) ranged from 89 to 563 mg/day and was generally adequate to fish consumption. The exception was for the Greek population which had a relatively higher fish consumption than EPA + DHA consumption, suggesting a predominant consumption of white non-greasy fish. Overall, participants in the European cohorts consumed more fish than the US cohorts [8].

As World Health Organization (WHO) experts concluded, promotion of a healthy lifestyle (including proper nutritional behavior) and prevention of diseases are the most effective and efficient ways to decrease premature mortality from non-communicable diseases (NCD) and to maintain mental health and well-being [10]. It is recommended to eat at least two servings of fish per week, one of which should be oily fish [2]. Seafood is of interest as a dietary component with potential beneficial effects in preventing the development of chronic NCD due to the presence of long-chain *n*-3 fatty acids (especially in oily marine fish), essential amino acids, vitamins and minerals and ingredients with antioxidant activity [11,12,13,14]. Considering the beneficial effects of these ingredients on blood pressure, lipid profile and the inflammatory process, seafood should be an essential component of the diet. Meanwhile, the consumption of this group of products varies globally. Several epidemiological studies have shown that higher fish consumption is associated with lower rates of cardiovascular diseases (CVD), coronary heart disease (CHD), and cerebrovascular mortality (including mortality from stroke, myocardial infarction and sudden cardiac death) [15,16,17].

The World Health Organization (WHO) estimates that the global elderly population will increase from 12% to 22% by 2050 [18]. It has been estimated that non-communicable diseases (NCD) such as cardiovascular diseases, cancer, diabetes and mental disorder, chronic respiratory diseases, which are common diseases associated with advanced age [19], are the leading cause of death in the world (over 70% of all deaths worldwide in 2016). Probability of premature mortality from NCD in the age group of 30–70 years old ranges between 8% and 31%, depending on geographical localization [10]. NCD are an economic burden on the economy, especially in developing countries [20].

Therefore, it is very important to introduce early prophylaxis in order to prevent age-related diseases, to minimize the risk or slow down their development, thus affecting well-being of older adults [19].

This narrative review briefly describes the nutrients and bioactive components found in seafood. It shows the potential preventive and therapeutic effects of seafood with an emphasis on fish based on 14 systematic reviews and meta-analysis published in 2007–2020. The systematic reviews and meta-analysis are considered to be the most powerful assessing tools [21].

## 2. Nutrients and Bioactive Compound

Seafood has long been considered a very valuable dietary component, mainly due to presence of *n*-3 polyunsaturated fatty acids (*n*-3 PUFA) but it is also an important source of protein (including collagen), anserine, taurine, iodine, selenium, vitamin A, vitamin K, vitamin D, tocopherols, B vitamins and astaxanthin [11,22,23,24,25,26].

### 2.1. Eicosapentaenoic Acid and Docosahexaenoic Acid

Eicosapentaenoic acid (EPA) and docosahexaenoic acid (DHA), which are included in the so-called long-chain acids (*n*-3 LC PUFA) have particularly health beneficial properties [22]. EPA and DHA acid show a beneficial effect on endothelial dysfunctions, anti-inflammatory properties [27,28,29,30], and reducing blood viscosity [31]. *N*-3 fatty acids lower the level of lipids [32], blood pressure [33] and decrease the risk of cognitive decline [34]. Vitamin D and selenium also present in fish, can enhance the neuroprotective effects of long-chain *n*-3 fatty acids [34]. Selenium (especially in the form of selenoproteins) and vitamin D are involved in several processes within central nervous system [35,36,37]. Vitamin D receptors are present in numerous tissues, including brain cells. Vitamin D is an important factor in regulation of the development and functioning of nerve cells. It probably plays a role similar to that of neurosteroids, influencing intracellular metabolism. The active form of vitamin D is involved in the synthesis and release of nerve growth factor (NGF—neurotrophic factors influencing neuron differentiation) and increases the levels of glial cell line-derived neurotrophic factor (GDNF). It also modifies the expression of (i) genes encoding the enzyme choline acetyltransferase (CAT) which participates in the synthesis of acetylcholine neurotransmitter, and (ii) genes associated with GABA-ergic neurotransmission. The hormonal form of vitamin D (1.25-(OH)2 D3) as well as selenium influence calcium ion pathways in the neuronal environment by altering the calcium ion homeostasis. Vitamin D activates the glutamyl transpeptidase enzyme activity and thereby stimulates the synthesis of glutathione. Selenium, selenoproteins and vitamin D protect the cells of the central nervous system against oxidative damage [36,37]. The long-chain docosapentaenoic acid (22:5*n*-3) is also present in seafood. It is believed that both the circulating and tissue level of this fatty acid may have a beneficial cardiovascular effect [38].

The richest sources of EPA and DHA are mainly fatty fish (including mackerel, salmon, herring, sardines, sprats, farmed trout). Depending on the fishing season, these fish contain from 1.4 to 2.5 g of EPA and DHA in 100 g of muscle tissue [22,39]. As a rule, the lowest EPA and DHA content in oily fish occurs during spawning (March–April) and winter migration, i.e., when the fish are not feeding [40]. However, the highest content occurs after the period of abundant foraging, which most often occurs in the autumn months, between September and October [39].

Farmed seafood is wrongly viewed as being nutritionally inferior to wild seafood. Taking into account EPA and DHA, farmed fish, both marine and freshwater, are often a richer source of fatty acids. This is due to the higher fat content of the feed, which is often based on fishmeal or fish oil rich in EPA and DHA. Farmed seafood also have a less intensive lifestyle compared to their wild counterparts, therefore their fat content is higher [41,42].

Interestingly, a decrease in the content of *n*-3 LC PUFA (DHA, EPA) in farmed fish has been observed for several years, as a result of replacing fishmeal and fish oil with vegetable oils in feeds [43,44].

Seasonal fluctuations in the content of lipids and *n*-3 PUFAs occur in both marine fish [45] and freshwater fish [46,47] and are related to fish development cycle (lipid metabolism) and food (availability and composition of the food) [48].

When comparing content of EPA and DHA in seafood, only absolute content of acids per 100 g of the product should be taken into account. The weight of 100 g of muscle tissue of farmed salmon contains 1.36 g of EPA and DHA, while in wild salmon—0.76 g. Observed differences result from higher lipid content in muscle tissue of farmed fish [43]. The lowest content of EPA and DHA, 80–160 mg/100 g, is found in lean fish (cod, pollock, hake) and mollusks such as octopus and cuttlefish.

Assuming that the daily requirement of DHA + EPA is 500 mg [49], it is enough to eat only 20–75 g of oily fish to provide this amount of acids. It does not seem to be a large amount, so it can be easily included in diet even for people who do not prefer fish dishes.

### 2.2. Astaxanthin and Tocopherols

Astaxanthin (AX) is a red pigment from the carotenoid group, which does not have the properties of provitamin A. The marine products such as alga, shrimp, crabs, trout, krill, lobster, crayfish, salmon and salmon roe are the source of AX [50,51]. It is used as a feed ingredient for farmed fish and as a food coloring [23]. Among salmonids, the content of astaxanthin ranges from 3 to 37 mg/kg and the highest content was estimated in free-living species where the content was at the level of 26–38 mg/kg flesh [23,52]. Thus, a 200 g serving of salmon provides about 1–7 mg of astaxanthin. In a randomized, double-blind, placebo-controlled trial conducted in healthy subjects aged 35–69 years, no side effects were found with a daily intake of 6 mg of astaxanthin (*H. pluvialis* algae extract) [53].

Among carotenoids, astaxanthin most effectively protects cells, lipids and lipoproteins of cell membranes against oxidative damage (fluorometric assay: BODIPY 665/676 or BODIPY 581/591 C11 as an indicators; AMVN as a peroxyl radical generator; Trolox as a calibrator). Astaxanthin was more effective than fish oil in modulating the immune system response and reducing the risk of vascular and inflammatory diseases. The antioxidant activity of astaxanthin is 10 times more than zeaxanthin, lutein, canthaxanthin, β-carotene and 100 times higher than α-tocopherol [23]. Oxidative stress and inflammation are pathophysiological features of atherosclerotic cardiovascular disease and cancer [54].

α-, β-, γ-, and δ-tocopherol, fat-soluble vitamin, which are present in seafood also have antioxidant properties [24]. The vitamin E content in fish is higher than that of meat or poultry and varies between species and tissues (higher amounts in dark muscle). The content ranges from 0.1 mg/100 g in some wild fish species to 3–4 mg/100 g in aquaculture fish and depends on diet, season, age, and size. For example, in cod, which is a lean fish, the level is 0.3 mg/100 g. In seafood the level of tocopherols decreases as a result of freezing storage for 6 months and of cooking [24].

The presence of other vitamins and selenium in seafood enhances the pro-health effect of tocopherols [55]. Vitamin E has been proposed for the prevention against colon, prostate and breast cancers, some cardiovascular diseases, ischemia, cataract, arthritis and certain neurological disorders [56].

### 2.3. Protein

In case of the lean fish, the main ingredients are proteins, which are a great source of amino acids (AA), including nutritionally essential (EAA), nonessential (NEAA), conditionally essential (CEAA), as well as functional amino acids (FAAs) [57,58]. The latter includes arginine, cystine, leucine, methionine, tryptophan, tyrosine, aspartate, glutamic acid, glycine, proline and taurine [58].

FAAs perform a variety of functions in the human body, including regulation of gene expression, cell signaling via kinase pathways, stimulation of brown adipose tissue development and thermogenesis, appetite and body composition, modulation of immune responses and prevention of infectious disease, reproduction, hormone secretion and endocrine status, antioxidative defense and removal of toxic substances, anti-inflammatory mechanisms, regulation of apoptosis and aging, neurological function and behavior (including neuroprotective effects), regulation of blood flow and cardiovascular function (e.g., NO synthesis) and recovery from injury. Accordingly, FAAs show great potential in the prevention and treatment of metabolic diseases, e.g., obesity, diabetes, cardiovascular disorders, intrauterine growth restriction, infertility, intestinal and neurological dysfunction, and infectious disease [58].

The content of these amino acids in different species of fish varies, resulting in the following range of content for arginine 0.1–6.5 g/100 g protein; leucine 0.1–10.4 g/100 g protein; methionine 0.02–4.0 g/100 g protein; tyrosine 0.03–8.4 g/100 g protein; tryptophan 0.1–6.5 g/100 g protein; cystine 0.03–0.6 g/100 g protein; aspartate 0.1–12.3 g/100 g protein; glutamic acid 0.2–16.5 g/100 g protein; glycine 0.1–13.7 g/100 g protein; proline 0.07–9.6 g/100 g protein [59].

Interestingly, a diet based on lean fish (such as cod) provides more certain amino acids (alanine, arginine, aspartic acid, glycine, methionine, and lysine) compared to a diet in which the lean fish is replaced with lean poultry, beef, veal or pork. In studies involving insulin-resistant men and women, it was found that cod protein diet improves insulin sensitivity compared with other lean animal protein sources. This effect is probably dependent on the amino acid composition, e.g., lower branched-chain amino acids (BCAAs) and higher arginine content [60]. An increase in sex hormone-binding globulin [61] and high-density lipoprotein 2 (HDL2) cholesterol concentrations [61,62] was also observed.

Collagen (mainly type I collagen) is the most abundant protein of intramuscular connective tissue in fish [63] and mostly is composed of amino acids such as glycine, valine, proline, and alanine [64]. Collagen obtained from fish skins, fish bones and scales is used in the pharmaceutical, cosmetic and food industries. Glycine has been shown to, in addition to reducing the symptoms associated with arthritis, have a beneficial effect on the growth of nails and hair [64].

### 2.4. Taurine and Anserine

Taurine is classified as an amino acid with antioxidant properties, but it is not built into the structure of proteins. Among foods, taurine is the most abundant in seafood (up to 800 mg/100 g in scallops), compared to 300 mg/100 g in turkey [65]. Cooking seafood in water causes its greatest losses [66].

Interestingly, in patients with metabolic syndrome, obesity, type II diabetes and cardiovascular diseases, lower plasma taurine concentrations were observed compared to healthy subjects. The decline in plasma and tissue taurine levels is also associated with the aging process [13].

The physiological function of taurine and its derivatives is very complex and has beneficial properties on many systems: cardiovascular, digestive, endocrine, immune, muscular, neurological, reproductive, and visual systems. Taurine provides antioxidant protection for cells and tissues, including the brain. As a component of bile salts, it is involved in the absorption of fat and fat-soluble vitamins and in the elimination of cholesterol via the fecal route. In addition, it stimulates the development of the nervous system and shows anti-inflammatory and antiapoptotic properties. The taurine derivative, *n*-chlorotaurine participates in defense mechanisms against pathogens: viruses, bacteria, fungi and parasites [11].

Anserine is a carnosine-like dipeptide [67] abundant in fish skeletal muscles e.g., salmon, tuna, trout [68]. For humans, it is an exogenous compound [69] and is metabolized into carnosine [70]. Physiological role includes H^+^ buffering, antioxidation and modulation of muscle contractility [68,70].

Human clinical trials have shown anserine to be beneficial for metabolic (reduced blood glucose), ageing-associated neurological (cognitive and memory), inflammation, immunological, cardiovascular and renal functions and also enhance muscular strength [11].

## 3. Potential Health Benefits

### 3.1. Cardiovascular Diseases and Mortality

Cardiovascular diseases are the leading cause of death in the United States. Mortality after the onset of heart failure (HF) is estimated at almost 50% within 5 years of the diagnosis [71]. As the incidence of obesity, diabetes and hypertension is increasing, the incidence of HF is projected to increase in the coming years. Coronary artery disease (CHD) and hypertension are the main causes of HF, therefore reduction of the risk of CHD and hypertension may reduce the incidence of HF [72].

Some studies have shown geographical differences in the effect of fish consumption on mortality risk. An inverse relationship has been observed in studies on the Asian population [73,74], while some Western studies have not shown a relationship and even reported a higher risk associated with high fish consumption [75,76,77].

Zhong et al., after 30 years of follow-up (mean follow-up 19 years) and a group of 29,682 American adults (mean ± standard deviation (SD) age was 53.7 ± 15.7 years at baseline), found that higher consumption of processed meat, unprocessed red meat or poultry but not fish, is significantly associated with a higher risk CVD occurrences [78]. The median (interquartile range (IQR)) intake in servings per week was 1.6 (0.9–3.4) for fish. The association between fish consumption and the incidence of CVD was stronger for participants who consumed significant amounts of protein in their diet than those who consumed less protein (HR, 0.96 (95% confidence interval (CI): 0.93–0.99) compared to 1.02 (95% CI: 0.99–1.05); *p* for interaction = 0.002) [68]. Additional intake of two servings of fish per week was not significantly associated with CVD incidence (HR, 1.00 (95% CI: 0.98–1.02); 30-years adjusted absolute risk difference (ARD), 0.12% (95% CI: −0.40% to 0.65%) and with all-cause mortality (HR, 0.99 (95% CI: 0.97–1.01); 30-years adjusted ARD, −0.34% (95% CI, −0.88% to 0.20%) [78]. There was no significant difference between the consumption of oily and non-oily fish with regard to CVD incidents and all-cause mortality [78]. However, in a 10-year follow-up with sensitivity analysis, the authors observed a statistically significant inverse relationship between fish consumption and total mortality (all causes of death) [78].

Two meta-analyses showed a weak inverse correlation between fish consumption and the risk of developing CVD or mortality [79,80]. Jayedi et al. analyzed 14 studies with 911 348 participants (age range 30–84; follow-up duration of 5–30 years) and found that increase of 20 g/day in fish intake was significantly and inversely associated with the risk of total CVD mortality and inversely associated with the risk of all-cause mortality. Interestingly, the shape of the association varied depending on geographical region (linear—Asian studies or U-shaped—Western studies) [79]. The association between 20 g/day increase in fish consumption and all-cause mortality was significant only among Asian studies (RR = 0.97; 95% CI: 0.96–0.98; *I^2^* = 0%, P_heterogeneity_ = 0.49, *n* = 5) compared to Western studies (RR = 0.99; 95% CI: 0.97–1.01; *I^2^* = 80.3%, P_heterogeneity_ < 0.0001, *n* = 8). It was also significant only in the subgroup of studies with follow-up duration <13 years (RR = 0.97; 95% CI: 0.96–0.99; *I^2^* = 57 5%, P_heterogeneity_ = 0.02, *n* = 8) compared to >13 years of follow-up (RR = 1.00; 95% CI: 0.98, 1.01; I^2^ = 76.7%, P_heterogeneity_ = 0.001, *n* = 6) [79]. A relatively rapid decrease in risk was observed with an increase in fish consumption of over ~60 g/day (P_non-linear_ < 0.0001) [79].

Djousse et al. [80] found that higher fish consumption and higher dietary or plasma EPA/DHA levels were associated with an approximately 15% lower risk of HF compared to the corresponding lower exposure category [80]. There is also evidence of a linear and inverse relationship between fish consumption and the risk of HF. There was a 5% lower risk of HF [RR: 0.95 (95% CI: 0.93–0.98)] with a higher fish consumption of 15 g/day (equivalent to one additional fish per week). In the pooled analysis between the highest versus lowest category of fish intake, a higher intake of fish was associated with a 15% (95% CI: 1% to 27%; *I^2^* = 8%) lower risk of HF. Sensitivity analysis based on geographical location, showed that in USA pooled RR is 0.69 (95% CI: 0.54–0.89). Heterogeneity has not been demonstrated [80].

In turn, Zhao et al. [81] showed a significant inverse relationship between the consumption of fish and the risk of all-cause mortality at the consumption of 60–80 g/day. Further increase of the amount did not affect the RR value [81].

### 3.2. Metabolic Syndrome and T2DM

Metabolic syndrome is a serious public health problem in Western countries. Its incidence has risen rapidly over the past two decades. According to data from the National Health and Nutrition Examination Survey (NHANES), approximately one-third of American adults suffer from this syndrome [82,83].

Meta-analysis of relative risk from three prospective cohort studies (7860 participants; 18–69 years old) indicated a significant inverse association between fish consumption and development of metabolic syndrome comparing the highest and the lowest category of intake (RR: 0.71, 95% CI: 0.58, 0.87; *I^2^* = 60.7%, *p* = 0.08). The increment of one serving of fish/week reduces metabolic syndrome risk by 6% (RR: 0.94; 95% CI: 0.90, 0.98; *I^2^*= 66.3%, *p* = 0.052) [84].

The meta-analysis of 16 studies (overall number of participants 679,763) with a minimum and maximum follow-up period of 4 and 23 years, respectively (11.5 years an average), indicated that seven times increase in fatty fish consumption reduces the risk of T2DM (RR 0.89; CI = 0.801, 0.987; *I^2^* = 0) [85]. The same study found no significant effect of lean fish and shell fish consumption on T2DM. Interestingly, among the Asians and Australians three times higher consumption of marine *n*-3 fatty acids reduces the risk of diabetes (RR 0.857, CI 0.79–0.93) [85].

### 3.3. Cancer

In particular, oily fish are a good source of *n*-3 fatty acids which may have anticancer properties against various types of cancer [86]. Meta-analysis by Tavani et al. [87] showed an inverse relationship between the consumption of *n*-3 polyunsaturated fatty acids and ovarian cancer [87]. However, the pooled analysis of fish consumption and cancer risk did not show such a trend.

The meta-analyses based on two case–control studies indicated an increased risk of endometrial cancer associated with total fish consumption (OR: 1.88; 95% CI: 1.20–2.98). However, as the authors note, the evidence is limited and inconsistent [88].

Kolahdooz et al. [86] in their meta-analysis defined “the total fish” as “canned tuna and dark-meat fish such as sardines (also classified as fatty fish), other types of fish (also classified as nonfatty fish), fish sticks, and seafood such as prawns and crabs” [86]. Analysis based on overall six studies (16 886 patients, at the age of 18–79 years old) such as population based case–control studies and hospital-based case-control studies showed an inverse relation between total fish consumption and the risk of ovarian cancer (RR: 0.84; 95% CI: 0.68, 1.03; P_heterogeneity_ = 0.003) [86].

Dose-response analysis of 13 cohort studies indicated that overall RR for the fish intake of 120 g/day was 1.07 (95% CI: 0.94–1.21) with moderate heterogeneity (*I^2^* = 33.3%), therefore null association between fish intake and risk of breast cancer was observed. Additionally, subgroup analysis showed a similar tendency taking into account factors such as age, menopausal status, region, duration of follow-up and study type [89].

Similar results were obtained by Zheng et al. [90] in meta-analysis of 11 studies (687,770 participants) estimating the pooled relative risk between the highest versus lowest category of fish intake (RR 1.03, 95% confidence interval 0.93 to 1.14; *I^2^* = 54%). Dose–response analysis also showed no association with risk of breast cancer for 15 g/day increase of intake (RR 1.00, 95% Cl 0.97 to 1.03) [90].

In pooled analysis with 52,683 patients (range 18–97 years old; 9–22 years of follow-up) Wu et al. did not observe a statistically significant relationship between seafood intake and the risk of prostate cancer regardless of stage or grade for the following consumption categories: below 5 g/day, 5–40 g/day and above 40 g/day (RR 1.04 (0.98–1.09); *I^2^* = 25%). No separate analysis was performed for lean and fatty fish [91].

Another meta-analysis (49,661 participants) showed no strong evidence of a protective influence of fish consumption on prostate cancer incidence (even in subgroup analyzes for factors such as race, fish type and method of preparation, grade and stage cancer). A statistically significant 63% lower risk was found for prostate cancer-specific mortality comparing the highest versus lowest categories of fish intake (RR: 0.37; 95% CI: 0.18, 0.74). However, the results were based on only four cohort studies and there was significant heterogeneity in the results between studies (test for heterogeneity P = 0.001) [92].

Colorectal cancer is the third most common cancer in men (10.0% of all cancer cases) and the second most common cancer in women (9.2% of all cancer cases) worldwide [93]. An 11% decrease of risk of colorectal cancer was observed for fish intake (RR for 100 g/day = 0.89 (95% CI = 0.80–0.99, *I*^2^ = 0%, P_heterogeneity_ = 0.52)) based on 11 studies [93]. The association of fish intake and colon or rectal cancer risk were not significant, with RR = 0.91 (Cl 0.80–1.03, *I*^2^ = 0%, P_heterogeneity_ = 0.76, 11 studies) and RR 0.84 (0.69–1.02; *I*^2^ = 15%, P_heterogeneity_ = 0.31, 10 studies), respectively [93].

### 3.4. Cognitive Impairment

Zhang et al. in meta-analysis defined standard serving as 105 g. The sample of 21941 participants (55–94 years old; 2–9.6 years of follow-up) from four independent cohorts were included; four trials and five trials reported the association between fish consumption and various risk of adverse cognitive outcomes, respectively, dementia and Alzheimer’s disease (AD).

Overall RRs and 95% confidence intervals (CIs) were one-by-one investigated for an increment of fish (one serving/week). A dose–response meta-analysis showed that risk of dementia and AD was significantly reduced for an increment of one serving/week, respectively, RR 0.95 (95% CI: 0.90, 0.99; *p* = 0.042, *I^2^*= 63.4%) and RR: 0.93; (95% CI: 0.90, 0.95; *p* = 0.003, *I^2^* = 74.8%). Both results had no publication bias.

Whereas, for participants who had no fish consumption, relative risks (RRs) of AD were 0.79 (95% CI: 0.66, 0.95), 0.74 (95% CI: 0.62, 0.89), and 0.71 (95% CI: 0.62, 0.81) for 2, 3 and 4 servings fish/week, respectively. However, no significant curvilinear association with risk of dementia was observed (*p* = 0.176) [94].

Recently published umbrella meta-analysis of 34 meta-analyses on prospective observational studies indicated additional 100 g/day increment in fish consumption was associated with a lower risk of all-cause mortality (summary relative risk SRR: 0.92; 95% CI: 0.87, 0.97), cardiovascular mortality (SRR: 0.75; 95% CI: 0.65, 0.87), coronary heart disease (SRR: 0.88; 95% CI: 0.79, 0.99), myocardial infarction (SRR: 0.75; 95% CI: 0.65, 0.93), stroke (SRR: 0.86; 95% CI: 0.75, 0.99), heart failure (SRR: 0.80; 95% CI: 0.67, 0.95), depression (SRR: 0.88; 95% CI: 0.79, 0.98), and liver cancer (SRR: 0.65; 95% CI: 0.48, 0.87) [95]. These outcomes were evaluated as moderate-quality evidence [95]. The results of described studies are summarized in Table 1.

## 4. Culinary Determinants of Seafood Health Properties

Thanks to the lipids, seafood has a widespread opinion that it is “good for everything”. Despite this opinion, there are studies showing a positive correlation between fish consumption and the risk of developing cancer of the prostate [96], stomach [97] or even colon [98]. Certain factors, including the method of preparing the fish, the types of fish consumed and the level of local contamination, may determine the impact of fish consumption on health outcomes in different regions [79]. Higher consumption of nonfried fish was inversely associated with the risk of mortality from coronary heart disease, while a nonsignificant trend towards higher risk was observed with increasing consumption of fried fish [99]. Another Australian prospective cohort study indicated that the consumption of uncooked fish was marginal and inversely associated with the risk of CVD mortality in women, while total fish consumption was not related to that risk [100]. There are also different types of fish to consider. Lean fish have lower amounts of *n*-3 fatty acids and are more often deep-fried [99]. Meanwhile, oily fish is generally high in *n*-3 and the results from a population cohort study in China suggest that higher consumption of oily fish may be more strongly associated with a lower risk of death from any cause and CVD compared to nonfatty fish [101]. It should be borne in mind that this correlation is the result of the formation of free radicals during intense heat treatments or the presence of salt that often accompanies seafood products. Both salt and some lipid oxidation products show a strong carcinogenic effect [102,103]. *n*-3 LC PUFA, EPA and DHA, in particular, are characterized by a high level of unsaturation, which promotes oxidation reactions. These reactions can take place both in the enzymatic and nonenzymatic way. The latter are induced mainly by free radicals.

Eating at least one serving a week of fried fish or shellfish (shrimp and oysters) results in a risk ratio for all-cause mortality of 1.07 (1.03 to 1.12) and 1,13 (1.04 to 1.22) for cardiovascular mortality in the US female population (aged 50–79 at study entry, *n* = 106,966) [104]. On the other hand, in another study involving the Spanish population, no adverse effects were observed [105]. This discrepancy is likely due to the fact that the effect is also dependent on the source of the fried seafood and the frying fat [104]. In the USA, fried food is most often bought, for example, in fast food restaurants, and is prepared in deep oil. Corn oil is commonly used for this purpose. In contrast, in the Mediterranean countries, both olive oil (home-cooked meals) and corn oil (meals prepared in the restaurants) are used for frying [104]. Therefore, it is extremely important to properly select ingredients and cooking methods for the preparation of seafood-based dishes.

The dishes should be prepared using thermal treatment, in which the heat is transferred by convection and/or radiation, i.e., boiling and baking. However, frying, in which heat is transferred by conductivity, should be avoided as it results in a sudden increase in the temperature of the outer parts of seafood [106]. In general, all cooking methods contribute to lipid oxidation [106], however, those in which heat is transferred by convection are characterized by a lower level of oxidation [107].

Elderly people are not advised to eat raw seafood [108], therefore, these dishes should be prepared in the mildest possible conditions. They should also avoid salted, dried and cold-smoked seafood because of its high salt content [14,102].

## 5. Seafood Safety

### 5.1. Pollution in Seafood

Environmental pollution has led to contamination of the fish mainly with dioxins and mercury in methylated form (MeHg). Most of the pollutants undergo biogenochemical migration accompanied by the process of bioaccumulation along the subsequent links of the food chain, e.g., in the muscles of fish. It poses a serious health risk to their potential consumers [109].

The adverse health effects associated with chronic exposure to dioxins include carcinogenic, immunotoxic, embryonic and fetotoxic, teratogenic and hepatotoxic effects [110]. In adults, the possible health effects associated with the consumption of MeHg is mainly the increase in oxidative stress. As a consequence, it may lead to, inter alia, atherosclerosis, myocardial infarction, heart rate variability and hypertension [110].

The literature clearly shows that the health benefits of consuming oily fish (2–3 servings per week) far outweigh the potential contamination risks [111,112]. In addition, EPA and DHA are known for their strong antioxidant, anti-inflammatory and pro-extinguishing properties, which mitigate the effects of exposure to such pollutants as polycyclic aromatic hydrocarbons (PAHs) [113], polychlorinated biphenyls (PCB) [114] and 2,3,7,8-Tetrachlorodibenzo-p-Dioxin (TCDD) [115]. There are even countries such as Finland, Sweden and Latvia which allow trade of fish in which permissible level of dioxins has been exceeded (Regulation of European Union (EU) 2016/1139). Tocopherols which are very often found in seafood can also reduce the effects of oxidative stress caused by pollution [116].

The risk related to the presence of contamination can be significantly reduced by choosing seafood from the appropriate fishing areas.

Although the levels of such pollutants as PAHs, dioxins, heavy metals and radionuclides in cod did not exceed the limits set by the EU, fish from the Baltic Sea had the higher content of contaminants compared to fish from the Barents Sea or Greenland [109].

Catch area is also of key importance in the case of mercury contamination in seafood. Depending on the geographical area, the mercury content of tuna varied from 0.03 to 0.82 mg/g [117].

Fish, such as shark and swordfish, which are at the top of the aquatic food chain, often have higher levels of contamination than other fish. That is why choosing the right species is so important to reduce consumption of pollutants. Research by Cammiller et al. [118] showed that the mercury content in bluefin tuna was nearly three times higher than in yellowfin tuna [118].

In addition to the catch area and species, factors such as the age and the season of fishing also play a significant role in the concentration of pollution in the seafood. Although the content of dioxins and dioxin-like compounds in Norwegian herring is generally low, the fish caught in the period between January and February had a higher content of contaminants compared to those caught in the period between April and June. Older specimens were also characterized by a higher accumulation of pollutants than younger specimens [119]. As in the case of lipids and *n*-3 PUFA, also in the case of contaminants, the physiological state of seafood related to its seasonality plays an important role.

In the spring the contaminants are transferred within herring body along with lipids from the muscle tissue to the gonads during their maturation which leads to decrease of their level. In addition, these fish can transfer also a certain amount of pollutants to their offspring via their gonads. In this way, they eliminate lipid-soluble impurities, thus reducing their toxic effects [119].

Contrary to the popular belief, the level of contamination in farmed seafood is at a similar level or often lower than the one of its wild counterparts [120,121]. Some publications show that wild fish have a lower content of contaminants than farmed fish [122,123]. However, the fat content is ignored in these studies. As it is commonly known, most contaminants are lipophilic, so the studies should include this variable [120]. For this reason, farmed fatty fish have a higher level of contamination compared to farmed lean fish [124,125]. In addition, the fat content within the fillet is not the same, which makes it difficult to compare the content of impurities if different parts of the fillet were analyzed, e.g., the dorsal or the abdominal [126]. Due to the fact that the content of contaminants in feed is strictly limited and controlled [127], this translates into a lower level of contamination in farmed fish. It is estimated that in the future, the level of contamination in salmon fillets will be close to the limit of quantification due to the development of methods of purifying fish oil and replacing it in part with plant ingredients [128,129].

### 5.2. Seafood Allergy

Certain food hypersensitivity may develop during adulthood. Examples are shellfish and fin fish allergy [130,131] and it may concern, respectively, 2.9% and 0.9% of the US adult population [132]. A meta-analysis showed that 0–2% of adults are hypersensitive to fish and 0–10% to crustaceans [131]. Average age of the adult seafood-allergic patient is 50 years old [1]. In adults over 60 years old, the prevalence of various seafood products allergy is similar to that in younger age groups [132]. Most often, elderly people report hypersensitivity after consuming shellfish (2.6%; 95% Cl:2.2–3.0), shrimp (1.6%; 95% Cl:1.3–1.9) and mollusks (1.2%; 95% Cl:1.0–1.5) [132]. The most commonly reported symptoms are skin, respiratory and gastrointestinal symptoms [1]. In Australia, seafood is the most common cause of fatal food anaphylaxis [133]. Ethnically, the most common seafood allergy is among Caucasians and African-Americans [1].

In the available literature there are controversial opinions about the relationship of radiocontrast, iodine, protamine and seafood allergies [134,135,136,137,138]. The major fish allergen is the low molecular-weight calcium-binding protein (parvalbumin). Allergy to seafood is not related to iodine. Iodinated contrast agents and povidone are the main iodinated drugs used in the radiology procedures and perioperative setting. IgE-mediated, both immediate and delayed hypersensitivities have been documented. However, the allergenic determinants remain unknown. The risk of reactions to radiocontrast ranges from 0.2% to 17%, severe reactions occur in 0.02–0.5% and deaths in 0.0006–0.006% [134]. A systematic review from seven prospective studies showed that the risk of reaction after radiocontrast media injections in patients with a seafood allergy is similar to that in patients with other food allergies or asthma [136,137]. Up to date, there is no evidence to avoid the use of iodinated drugs in seafood allergy.

Protamine is a polypeptide, isolated from salmon fish sperm. It is also used in insulin preparation to prolong the pharmacological effect. The evidence for an IgE-mediated allergy to protamine is very limited. Evidence supporting the increased risk for protamine allergy in fish allergy is lacking [136]. Up to date, there is no evidence to avoid the use of protamine or NPH insulin in fish allergies [134,136,137].

## 6. Conclusions

This review presented a broad overview of the association of seafood (especially fish) intake with the risk of non-communicable disease based on systematic review and meta-analysis. Although some of the results clearly do not state an impact on health, our review has important implications and indicates the direction of future research.

It is essential to evaluate the impact of intake of different types of fish such as fatty, lean, processed (e.g., smoked, canned, salted fish), methods of cooking fish (e.g., frying, steaming) on health and what potential factors cause these differences in effect in presented studies.

On the other hand, this strong or moderate evidence of the beneficial effects of seafood suggests that promoting a diet based on the regular consumption of different types of seafood should be further strengthened in populations of all ages. However, to get the full benefits of eating seafood, it should be cooked properly so as not to lose its health benefits. Our present review provides evidence that seafood, especially fish intake, indicate the potentially beneficial effects of the antiaging process and well-being in the elderly population. Taking action to reduce the incidence of non-communicable diseases in the elderly group (primarily changing the lifestyle, including nutritional behavior) is a moral and an economic imperative.

## Figures and Tables

**Table 1:** Summary of significant potential health benefits of fish consumption in previous meta-analysis.

References	Studies (*N*)	Participants (*n*)	Outcome	RR (95% Cl)/SRR *
Jayedi et al. (2018) [79]	14	911,348	All-cause mortality	0.97 (0.96–0.98)
Djousse et al. (2012) [80]	5	170,131	Heart failure	0.95 (0.93–0.98)
Zhao et al. (2016) [81]	12	672,389	All-cause mortality	0.94 (0.90–0.98)
Kim et al. (2015) [84]	9	7860	Metabolic syndrome	0.71 (0.58–0.87)
Muley et al. (2014) [85]	16	679,763	T2DM	0.89 (0.801–0.987)
Kolahdooz et al. (2010) [86]	6	16,886	Ovarian cancer	0.84 (0.68–1.03)
Bandera et al. (2007) [88]	5	10,543	Endometrial cancer	1.88 (1.20–2.98)
Wu et al. (2016) [89]	13	758,359	Breast cancer	1.07 (0.94–1.21)
Zheng et al. (2013) [90]	11	687,770	Breast cancer	1.03 (0.93–1.14)
Wu et al. (2016) [91]		52,683	Prostate cancer	1.04 (0.98–1.09)
Szymański et al. (2010) [92]		49,661	Prostate cancer	0.37 (0.18–0.74)
Vieira et al. (2017) [93]	11	3944	Colorectal cancer	0.89 (0.80–0.99)
Zhang et al. (2016) [94]	4	21,099	Dementia	0.95 (0.90–0.99)
	5	21,941	Alzheimer’s disease	0.93 (0.90–0.95)
Jayedi et al. (2020) [95]	38	153,998	All-cause mortality	0.92 * (0.87–0.97)
	8	11,720	Cardiovascular mortality	0.75 * (0.65–0.87)
	22	16,732	Coronary heart disease	0.88 * (0.79–0.99)
	11	8468	Myocardial infarction	0.75 * (0.65–0.93)
	20	14,360	Stroke	0.86 * (0.75–0.99)
	8	7945	Heart failure	0.80 * (0.67–0.95)
	8	5732	Depression	0.88 * (0.79–0.98)
	5	1572	Liver cancer	0.65 * (0.48–0.87)

* SRR—summary relative risk. Type 2 Diabetes Mellitus (T2DM).
